# Field vaccination against CCHFV and *Hyalomma* tick infestation reduces multiple tick-borne infections in sheep

**DOI:** 10.1038/s41541-025-01367-8

**Published:** 2026-01-16

**Authors:** Moufid Mhamadi, George Giorgi Babuadze, Aminata Badji, Jose Echanove, Alioune Gaye, El Hadji Ndiaye, Oumar Ndiaye, Mignane Ndiaye, Idrissa Dieng, Ara XIII, Moundhir Mhamadi, Cheikh Talibouya Touré, Mathioro Fall, Ousmane Faye, Hugues Fausther-Bovendo, Oumar Faye, Amadou Alpha Sall, Gary Kobinger

**Affiliations:** 1 Virology department, The Pasteur Institute of Dakar, Dakar, Senegal; 2 Preclinical Studies Department, Vaccine Research Center, Institut Pasteur de Dakar, Dakar, Senegal; 3 University of Texas Medical Branch, Galveston, TX USA; 4 Medical Zoology department, The Pasteur Institute of Dakar, Dakar, Senegal; 5 Microbiology-Infectiology and Immunology department, Université Laval, Quebec City, QC Canada; 6 DIATROPIX, the Pasteur Institute of Dakar, Dakar, Senegal; 7 Ministry of livestock and animal production of Senegal, Dakar, Senegal; 8 Global Urgent and Advanced Research and Development, Batiscan, QC Canada

**Keywords:** Diseases, Immunology, Microbiology, Zoology

## Abstract

Tick-borne pathogens (TBPs) are expanding globally, with their impact on public health expected to rise due to climate change. Immunizing livestock offers a cost-effective alternative or adjunct to human vaccination. We evaluated two DNA vaccines, one targeting Crimean-Congo hemorrhagic fever virus (CCHFV) and another targeting *Hyalomma* tick infestation. The *Hyalomma*-targeting vaccine was designed to disrupt tick feeding by targeting midgut proteins essential for blood digestion and survival; however, its direct role in preventing CCHFV transmission remains unconfirmed. Here, we demonstrate that two doses of the CCHFV vaccine significantly reduced the risk of CCHFV infection in naturally exposed sheep. We further investigated whether the *Hyalomma* vaccine provided cross-protection against Wad Medani virus (WMV) and *Rickettsia conorii*, two TBPs endemic to Senegal. Sheep were vaccinated intramuscularly with two doses of DNA vaccine, followed by electroporation, and monitored under natural farming conditions in an endemic region of Senegal. Natural infection with CCHFV, WMV, and *R. conorii* was assessed longitudinally using pathogen-specific IgG seroconversion as the primary endpoint. The *Hyalomma* vaccine reduced WMV acquisition, whereas its effect on *R. conorii* was less pronounced. These findings underscore the potential of veterinary vaccines to mitigate multiple TBPs and reinforce their established role in reducing tick-borne diseases.

## Introduction

Human vaccine development for emerging pathogens is often slowed by the high cost of clinical evaluation^1^. The advancement of vaccines, both for human and veterinary use, faces several challenges, including regulatory requirements, the cost of research and development, limited profit margins, public perception, and potential legal liabilities arising from adverse or side effects. Veterinary trials can be particularly expensive, requiring millions of dollars in funding due to the need for specialized containment facilities for high-risk veterinary pathogen trials. Furthermore, the great majority of emerging pathogens causing diseases in humans are zoonotic. Immunization of reservoir species or species involved in spillover events into the human population could reduce the occurrence of zoonotic diseases. Reservoir-targeted vaccination strategies have been successfully employed for several zoonotic pathogens beyond rabies, including Lyme disease^2,3^, bovine tuberculosis, and plague. Recent studies highlight the broad applicability of this approach in controlling diseases at the animal-human interface^4,5^.

In recent decades, ticks have expanded their geographical distribution and prevalence. The geographic expansion of ticks and the introduction of invasive tick species further complicate vector control efforts. Climate change, land-use changes, and increased livestock trade have contributed to the spread of tick populations into new regions, leading to the emergence and re-emergence of tick-borne diseases^6^. As a result, the occurrence of tick-borne pathogens (TBPs) in the human population has increased. Countermeasures against TBPs, therefore, are needed to cope with the continuous rise in infections. Ticks are obligate ectoparasites that often become infected by and spread TBPs during blood meals on susceptible species. Thus, limiting ticks’ feeding on susceptible hosts could reduce the spread of TBPs. Acaricides are pesticides typically used to treat and prevent infestation in animals^7,8^. Beyond acaricides, integrated tick management (ITM) strategies, including biological control using entomopathogenic fungi, habitat modification to disrupt tick-host interactions, and genetic control approaches, offer additional methods for controlling tick populations while reducing environmental contamination^9–11^. However, tick resistance is a concern, as well as contamination of both the environment and the animal meat and dairy, as these chemicals could be detrimental to animal and human health^12,13^.

There exists a robust body of literature on anti-tick vaccines, dating back to the earliest studies in 1979, when Allen and Humphreys demonstrated that immunization with tick antigens could reduce tick infestation^14^. To date, two commercial vaccines, Gavac and TickGARD, have been developed to reduce infestation by *Boophilus microplus* ticks, based on the original characterization and field evaluation of the Bm86 antigen^15,16^. Both vaccines encode the Bm86 gut antigen and have demonstrated reduced infestation by *B. microplus* and *B. annulatus* in multiple field evaluations^17^. Additionally, they have been shown to lower the frequency of babesiosis, a parasitic infection transmitted by these tick species^18,19^. However, Gavac and TickGARD did not protect against *Amblyomma cajennense*, another tick species, suggesting that genus-specific Bm86-based vaccines may be needed to control tick infestation^20^. In contrast, a 20-amino-acid-long peptide derived from the acidic ribosomal protein P0 is highly conserved among ticks, while divergent from its bovine ortholog. Vaccination with the ribosomal peptide P0 reduced infestation of cattle and rabbits with *Rhipicephalus* ticks^21,22^. These studies highlight the potential and limitations of current anti-tick vaccines, emphasizing the need for further research into broader cross-protective approaches.

Crimean-Congo hemorrhagic fever virus (CCHFV) is a TBP on the World Health Organization (WHO) list of high-consequence pathogens of concern, for which only limited countermeasures are available^22^. CCHFV can cause severe hemorrhagic fever and death in humans^23^. An inactivated CCHFV vaccine, derived from suckling mice intracranial infection, is currently licensed in Bulgaria^24^. Due to its limited immunogenicity and the many safety concerns associated with manufacturing the vaccine in mice brains prior to inactivation, this vaccine has remained of low utility worldwide^25^.

Notably, immunization using mouse brain tissue has been associated with the development of targeted immune responses against nervous system components, raising significant safety concerns. CCHFV has been detected in numerous tick species; however, *Hyalomma* ticks are the main vector for CCHFV transmission^26,27^. In some settings, alternative transmission routes, such as direct contact with infected blood or tissues, may contribute more significantly to human infections than tick bites^28^. In Africa, livestock is an important contributor to the spread of CCHFV between animals and humans. Livestock can bring infected ticks within the community and frequently serve as a source of exposure for farmers. Once infected, animals can transmit the disease to individuals caring for or butchering them, as well as infect new tick populations during a blood meal, increasing tick density and CCHFV prevalence^29,30^. Multiple serological and entomological surveys have confirmed active CCHFV circulation in livestock, humans, and ticks in Senegal. Previous studies from the Matam and Agnam regions reported CCHFV-positive ticks, including *Hyalomma* and *Rhipicephalus* species, as well as seropositive livestock and human samples, indicating sustained endemic transmission in northern Senegal^29,30^. Notably, infected livestock do not exhibit clinical symptoms, making detection and control efforts more challenging^29^. Given that *Hyalomma* ticks serve as the primary vector for CCHFV, reducing tick infestations in livestock could indirectly reduce viral transmission^26,27^. Reducing tick populations has been shown to decrease tick–host contact rates and thereby lower transmission of multiple tick-borne pathogens^7,8^. Immunization of livestock against CCHFV or *Hyalomma* tick infestation, therefore, could reduce CCHF prevalence in Africa. Across Africa, seroprevalence of CCHFV in livestock typically ranges from 5% to over 40%, reflecting widespread viral circulation across East, West, and Southern Africa^26,27^.

This study documents the development and immunogenicity of two veterinary vaccines: one targeting *Hyalomma* tick infestation and another targeting CCHFV. We evaluated their protective efficacy in sheep against natural CCHFV infection in Senegal. Additionally, we assessed the impact of the *Hyalomma* vaccine on infestation rates and its potential to reduce transmission of WMV and *Rickettsia conorii*, two TBPs endemic to the region.

## Results

### Design and immunogenicity of the CCHFV and *Hyalomma* DNA vaccines

**Fig. 1:**
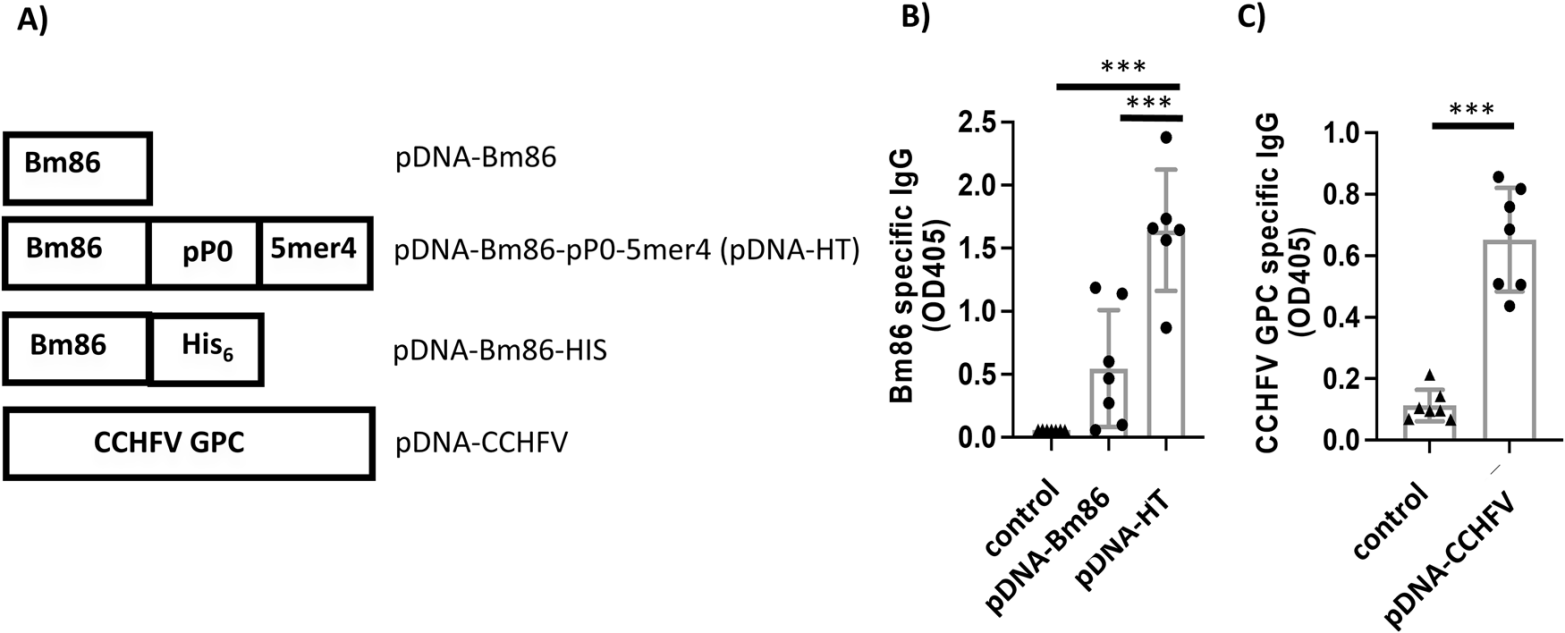
DNA vaccine constructs targeting Hyalomma tick infestation and Crimean–Congo hemorrhagic fever virus (CCHFV). Schematic representation of the different vaccine constructs developed (**A**). BALB/c mice were immunized with PBS or 100 µg per mouse of pDNA-Bm86, pDNA-Bm86-pP0-5mer4 (pDNA-HT), or pDNA-CCHFV. Vaccines targeting tick infestation were administered intramuscularly, while mice receiving pDNA-CCHFV were immunized intramuscularly followed by electroporation (IM-EP), as described in the Methods (**B**, **C**). The humoral responses against Bm86 (**B**) and CCHFV GPC (**C**) were measured by ELISA in sera collected 28 days after the booster dose and tested at 1:1600 (Fig. 1B) and 1:400 (Fig. 1C) dilutions, respectively. CCHFV-specific IgG responses were assessed using VLPs expressing the glycoprotein precursor (GPC) as the ELISA antigen. For each group (*n* = 7), the mean ± SEM is shown. *** indicates *p* < 0.001. CCHFV GPC refers to the full-length glycoprotein precursor encoded by the viral M segment, which is processed into the Gn and Gc envelope glycoproteins.

We hypothesized that vaccines against CCHFV or against *Hyalomma* tick infestation could reduce CCHFV transmission to livestock. Existing vaccines against *Boophilus* tick infestations encode the gut antigen Bm86^12^. Therefore, we cloned a codon-optimized Bm86-like antigen (the *Hyalomma* BM86 homolog) into a plasmid DNA expression vector. A second vaccine construct containing both a 20mer peptide from the ribosomal protein P0 (pP0) and the immunostimulatory peptide 5mer4 downstream of Bm86 was also engineered. The two constructs were denoted as pDNA-Bm86 and pDNA-Bm86-pP0-5mer4, respectively (Fig. 1A).

The 5mer4 peptide, a rare-occurring 5-amino-acid-long peptide, was previously shown to increase the immunogenicity and efficacy of an influenza vaccine when added downstream of the hemagglutinin (HA) protein^31^. The P0 ribosomal peptide was selected as it is highly conserved among ticks and was previously used as an immunogen to prevent tick infestation on various animals^17,18^. For the CCHFV vaccine, the full-length glycoprotein precursor (GPC) encoded by the viral M segment was cloned into the same plasmid backbone, and the resulting construct was designated as pDNA-CCHFV.

No detectable IgG antibody against the Bm86 protein was observed in control mice, while 4 of the 7 mice that were vaccinated with pDNA-Bm86 generated an IgG antibody response against Bm86 with an optical density (OD) value above 0.45. All mice (6/6) immunized with pDNA-Bm86-pP0-5mer were seropositive, with IgG antibody levels against Bm86 with OD above 0.9 observed at a 1:1600 dilution of the tested sera. The threshold OD value of 0.45 was determined as the mean OD value of negative control samples plus three standard deviations. This cutoff was established using sera from unvaccinated mice as a baseline reference for background reactivity. Samples exceeding this OD value were considered seropositive for an immune response against Bm86.

The Bm86-pP0-5mer4 construct was selected for further analysis, as Bm86-specific IgG levels in this group were significantly (*p* < 0.001) higher than for pDNA-Bm86 vaccinated mice (Fig. 1B). The Bm86-pP0-5mer4 construct was thereafter denoted as pDNA-HT.

The pDNA-CCHFV vaccine also generated humoral response in immunized mice, with an average OD of 0.65 (Fig. 1C). Overall, both pDNA-CCHFV and pDNA-HT were immunogenic and were further evaluated.

### In vivo efficacy of CCHF and *Hyalomma* tick vaccine

Next, we investigated whether the pDNA-CCHFV and pDNA-HT vaccines could protect animals against CCHFV infection in their natural environment in Africa, where CCHF is endemic. To that end, 173 local sheep from the Matam region in Senegal were selected for a vaccine study.

IgG antibodies against the CCHFV nucleocapsid (N) protein were detected in 20 of the 173 tested animals (11.6%), confirming the presence and spread of CCHFV in the area. No active CCHFV infection was detected by RT-qPCR in any of the tested sheep.

A total of 104 CCHFV-negative sheep were selected and divided into three groups: an unvaccinated control group (*n* = 34), a pDNA-HT-vaccinated group (*n* = 35), and a pDNA-CCHFV-vaccinated group (*n* = 35) (Table 1). Except for the control group, all animals received 1 mg of DNA intramuscularly followed by electroporation on day 0, with a second dose administered one month later. Animals were bled 14, 28, 42, and 56 days after the first vaccination and then monthly thereafter for 1 year to monitor seroconversion and natural CCHFV infection. Of note, 2 animals in the control and 1 from the pDNA-CCHFV vaccinated group died during the experiment and were excluded from subsequent analyses (Table 1).

**Table 1:** Sheep distribution within vaccine groups

	Sex			Age (months)			Death
	M	F	total	Min	Max	Median Age	*n* (day post vaccination)
Control	2	32	34	4	18	8	2 (28 and 206)
pDNA-HT vaccine	10	25	35	4	15	8	0 (N.A.)
pDNA-CCHFV vaccine	3	32	35	4	15	7	1 (86)

Local sheep without prior CCHFV exposure were divided into three groups. The sex and age distribution are indicated for the three experimental groups. The number of deaths that occurred during the study is also reported. N.A., not applicable.

**Fig. 2:**
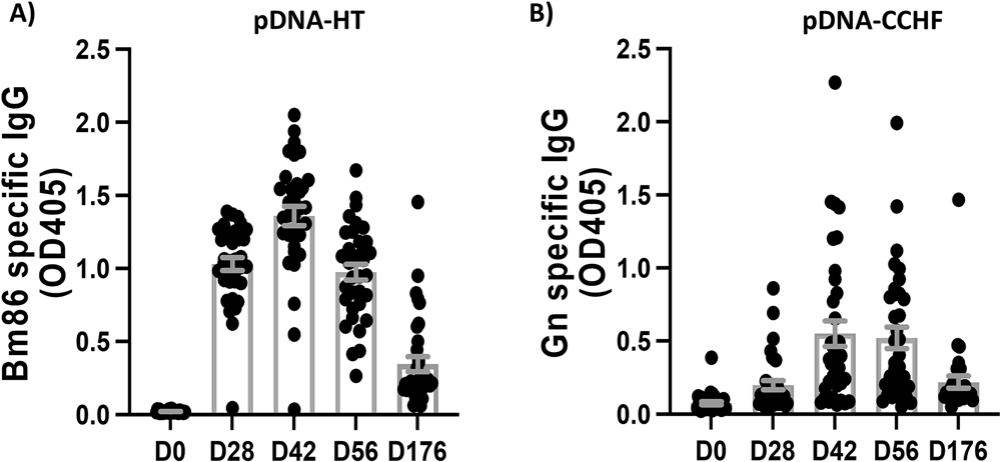
The immunogenicity of the pDNA-HT plasmid in sheep in a natural setting. The humoral response against Bm86 (**A**) and CCHFV Gn (**B**) was measured by ELISA in pDNA-HT (*n* = 35) and pDNA-CCHFV (*n* = 34) immunized sheep after two 1 mg intramuscular doses of DNA vaccine followed by electroporation (IM-EP) using the CELLECTRA-3P device, as described in the Methods, the mean ± SEM is depicted. Next, the protective efficacy against CCHFV infection of both the pDNA-HT and pDNA-CCHFV vaccines was analyzed. The development of IgG antibodies against the CCHFV N protein (OD > 0.223) was used as a surrogate marker for natural infection, as N is not encoded by the DNA vaccine constructs. In the control group, IgG antibodies against the CCHFV N protein were detected in 17 of 32 animals (53.1%). In contrast, only 5 of the 34 animals (14.7%) that received the pDNA-CCHFV vaccine developed N-specific antibodies. In the pDNA-HT immunized group, 13 of 35 animals (37.1%) showed antibody levels indicative of CCHFV infection, with 11 cases occurring on or after 176 days post–first vaccination (i.e., 148 days after the booster; Fig. 3). Overall, immunization with the pDNA-CCHFV vaccine significantly reduced CCHFV acquisition (*p* = 0.001), whereas the protective effect of pDNA-HT did not reach statistical significance (*p* = 0.19). The acquisition of Wad Medani virus (WMV) infection was evaluated over the 386-day follow-up period. An IgG response against WMV (OD > 0.231) was used as the threshold for natural infection. Among the sheep in the combined control and pDNA-CCHFV groups, 12 of the 31 animals monitored became infected with WMV. In contrast, WMV infection was detected in only one of the 35 animals in the pDNA-HT group, indicating a significant protective effect of the pDNA-HT vaccine (*p* = 0.0002; Fig. 3). Similarly, the pDNA-HT vaccine led to a reduction of R. conorii infection, from 93.8% (30/32) in the control group to 68.6% (24/35) in pDNA-HT immunized animals (*p* = 0.0001; Fig. 3). Taken together, these data suggest the pDNA-HT vaccine can protect against multiple TBPs.

The humoral response against Bm86 generated in pDNA-HT vaccinated sheep was measured by serology. All animals except one developed a strong response against Bm86. Overall, the magnitude of the Bm86-specific IgG response peaked around 42 days after the first immunization (i.e., 14 days after the booster) and declined thereafter (Fig. 2A). In pDNA-CCHFV–immunized animals, the humoral response against CCHFV peaked between days 42 and 56, with average OD values (mean ± SEM) of 0.55 ± 0.09 and 0.52 ± 0.07, respectively (Fig. 2B).

**Fig. 3:**
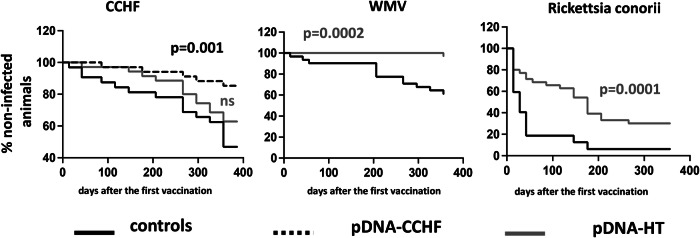
Protective efficacy of the pDNA-CCHFV and pDNA-HT plasmid in sheep under a natural setting. Kaplan–Meier curves showing the proportion of non-infected animals over time for CCHFV (left), WMV (middle), and *R. conorii* (right). All immunized animals received two 1 mg doses of plasmid DNA via the IM-EP route. For the CCHFV panel (left), unvaccinated animals (*n* = 33), pDNA-CCHFV (*n* = 34), and pDNA-HT (*n* = 35) groups were monitored longitudinally for the appearance of anti-N IgG as evidence of natural infection. For the WMV and *R. conorii* panels (middle and right), animals from the control (n = 32) and pDNA-HT (*n* = 35) groups were followed for the development of pathogen-specific IgG, indicating field-acquired infection. The X-axis represents days after the first vaccination, and the Y-axis shows the percentage of animals remaining non-infected at each time point.

## Discussion

TBPs, including CCHFV, are growing global health concerns and effective countermeasures are needed. The developed pDNA-CCHFV vaccine significantly reduced the risk of CCHFV infection in sheep kept under natural farming conditions in Senegal. This is significant considering that livestock are a major amplifying species for CCHFV, which can cause severe hemorrhagic fever in humans^32^. The pDNA-HT vaccine did not provide significant protection against CCHFV infection in vaccinated sheep. This observation may be related to the fact that, in the Matam region, CCHFV has so far been detected primarily in ticks of the ***Rhipicephalus*** genus^29^. At present, we are not aware of systematic comparative data on CCHFV prevalence in ***Hyalomma*** versus ***Rhipicephalus*** ticks from this specific area, and therefore, this interpretation should be viewed with caution. In sheep, the pDNA-HT vaccine reduced the likelihood of both WMV and *R. conorii* infection. Because this study relied on natural field exposure, all infections occurred through tick feeding under real-world ecological conditions. Pathogen circulation differs substantially among CCHFV, WMV, and *Rickettsia conorii* in Northeastern Senegal, resulting in variable exposure pressure. Thus, variation in acquisition frequencies likely reflects underlying pathogen prevalence rather than differences in vaccine activity. Importantly, the observation that pDNA-HT reduced the acquisition of multiple pathogens, despite their distinct ecological dynamics, highlights the broader potential of anti-tick vaccination as a cross-protective vector-targeted strategy. To our knowledge, this is among the first reports showing that a single tick vaccine can induce protection against a tick-borne virus and a tick-borne bacterium in farm animals exposed naturally to infection. By naturally exposed to infection, we refer to pathogen acquisition through routine tick bites occurring under field grazing conditions. Sheep remained in an endemic area where exposure to *Hyalomma* and *Rhipicephalus* ticks is continuous. Although CCHFV, WMV, and *R. conorii*-infected ticks have been detected in the region, the precise prevalence of infected ticks and the probability of an individual sheep encountering an infected tick cannot be directly estimated due to the heterogeneous and seasonally dynamic nature of tick-borne pathogen circulation. Its efficacy against other *Hyalomma*-transmitted pathogens requires further evaluation. Further studies will be required to evaluate the impact of the pDNA-HT vaccine on tick biology (feeding, fecundity). In addition, optimization of the vaccination schedule, particularly the boosting strategy, will be necessary. A third dose could enhance protection for long-term benefits, as a decrease in protection was generally observed approximately 5 months post-immunization. Of note, CCHFV and *R. conorii* transmission by non-*Hyalomma* ticks cannot be ruled out, as both pathogens can be found and are transmitted by several tick species^27,28,33^. Vaccines against multiple tick species could substantially expand protection^12^.

In veterinary settings, tick infestation has been traditionally controlled using acaricides, which over time leads to selection of resistant ticks in addition to creating toxicity concerns associated with the use of these chemicals in the food chain^12,13^. In contrast to acaricides, vaccines are unlikely to create resistance in ticks, especially if multiple genes are included, as is the case in the pDNA-HT prototype against *Hyalomma* infestation. Efficacious vaccines against TBPs could therefore offer a sustainable alternative that protects farming communities and micro-economies that provide food and income to millions of individuals.

Here, we documented the efficacy in real-life conditions, of two veterinary vaccines against CCHFV and TBPs in general. These vaccines show promise in reducing CCHFV infection risk and pathogen acquisition, including WMV and *R. conorii*. However, direct assessment of tick infestation was not conducted in this study, and further experiments are required to evaluate the vaccine’s impact on tick burden. While reductions in pathogen acquisition were observed, controlled challenge trials are needed to confirm protection against WMV and *R. conorii*. Regulatory assessments and manufacturing scale-up are also necessary before these vaccines can be considered for widespread implementation.

In summary, the pDNA-CCHFV vaccine significantly reduced natural CCHFV acquisition in sheep under endemic field conditions, while the pDNA-HT vaccine reduced infection with WMV and moderately decreased *R. conorii* acquisition. These findings support the utility of veterinary DNA vaccines as tools for mitigating multiple tick-borne pathogens in endemic regions. Further studies are warranted to assess optimal boosting intervals, effects on tick burden, and broader cross-protection against additional TBPs.

## Methods

### Anesthesia and euthanasia

For mouse experiments, animals were deeply anesthetized using isoflurane inhalation anesthesia (3–5% for induction and 1.5–3% for maintenance) prior to terminal procedures. Euthanasia was performed by cardiac exsanguination under deep anesthesia, followed by cervical dislocation, in accordance with approved institutional animal care protocols.

For sheep, intramuscular DNA vaccination followed by electroporation was performed without anesthesia, as sedation or anesthesia was not required for the procedure. Animals were manually restrained and handled by trained personnel according to local veterinary practices. No planned euthanasia was performed in sheep as part of the study; deaths that occurred during the study were incidental and unrelated to experimental procedures.

### Plasmid production

Tick-vaccine plasmids (pDNA-Bm86, pDNA-Bm86-pP0-5mer4, and pDNA-Bm86-HIS) were generated from gene fragments encoding bovine codon-optimized amino acid (AA) 1-646 from the BM86-like protein from *Hyalomma anatolicum* (AAL36024.1), either alone or followed by AA 288–302 from the ribosomal protein P0 (ALJ02546.1) and the 5mer4 peptide ^21,31^. were synthesized by GenScript (Piscataway, NJ) (Fig. 1A). Synthesized gene fragments were cloned into the plasmid DNA backbone using classical cloning techniques to generate pDNA-Bm86 and pDNA-Bm86-pP0-5mer4 (pDNA-HT).

Because no Bm86 antigen from *Hyalomma* species was commercially available, the Bm86 homolog was synthesized with incorporation of a HIS tag to facilitate protein purification and detection. The resulting plasmid construct (pDNA-Bm86-HIS) was validated by Sanger sequencing prior to experimental use. HEK293T cells were seeded in 24-well plates and transfected with the pDNA-Bm86-HIS plasmid using polyethylenimine (PEI, Polysciences, France) at a 1:3 plasmid-to-PEI ratio, following the manufacturer’s instructions. Supernatants were collected 3 days post-transfection and clarified by centrifugation at 8000 × *g*. HIS-tagged Bm86 protein was purified using nickel (Ni-NTA) agarose resin (Qiagen, Toronto, Canada) according to the manufacturer’s protocol, and protein purity was assessed by SDS-PAGE.

For the CCHFV vaccine, glycoproteins were targeted due to their role in viral entry and immune system recognition. Accordingly, gene fragments encoding a codon-optimized CCHFV glycoprotein precursor (GPC; ALT31694) were synthesized by GenScript and cloned into the same plasmid DNA backbone using classical cloning techniques ^34^ (Fig. 1A).

CCHFV virus-like particles (VLPs), using glycoproteins from the IbAr 10200 strain, were generated as previously reported^34,35^. VLP production was independent of the pDNA-CCHFV vaccine construct and relied on a published plasmid system expressing CCHFV IbAr10200 strain glycoproteins together with additional structural and replication components (pC-M Opt, pC-N, L-Opt, T7-Opt, and a NanoLuc minigenome plasmid). Briefly, HEK293T cells were transfected with these plasmids using FuGENE HD transfection reagent (Thermo Fisher Scientific, Burlington, Canada) according to the manufacturer’s instructions. VLPs were pelleted from clarified supernatants through a 20% sucrose solution (130 mM NaCl, 20 mM HEPES, pH 7.4) by centrifugation for 2 h at 106,750 × *g* at 4 °C. VLPs were resuspended overnight at 4 °C in 130 mM NaCl and 20 mM HEPES (pH 7.4) and stored at −80 °C in single-use aliquots.

### Mice immunization

Female 5–6-week-old BALB/c mice (Charles River, Quebec, Canada) were acclimatized for one week prior to the start of the study and housed under pathogen-free conditions (22 ± 2 °C; 12-hour light/dark cycle) with ad libitum access to food and water.

To evaluate vaccine-induced immune responses, mice were allocated into groups of seven animals each, receiving two intramuscular injections of 100 µg of pDNA-Bm86, pDNA-HT, or pDNA-CCHFV administered 28 days apart, with electroporation (CELLECTRA-3P, Inovio, San Diego, CA) applied immediately after each pDNA-CCHFV injection. Electroporation consisted of three pulses of 0.2 A constant current, each lasting 52 ms with 3-second intervals, delivered through a 3-needle electrode inserted approximately 3–5 mm into the tibialis anterior muscle. Mock-vaccinated mice received PBS. Blood samples were collected 28 days after the booster dose by cardiac puncture, and serum was separated by centrifugation and stored at −80 °C until use.

All animals were euthanized at this terminal time point. Humoral immune responses against Bm86 and CCHFV glycoproteins were quantified by enzyme-linked immunosorbent assay (ELISA), using in-house-produced Bm86 protein and CCHFV VLPs as coating antigens (Fig. 1B, C).

### Animal selection study site, sheep selection, and immunization protocol

This study was conducted in the Matam region of Senegal, where infestation by *Hyalomma* ticks is highly prevalent, and a variety of tick-borne pathogens (TBPs) are endemic. However, *Rhipicephalus* ticks are also present in this region and have likewise been reported to carry CCHFV^26,30^. This region is characterized by a semi-arid climate, with a high density of livestock farming, which contributes to the spread of tick-borne diseases. A total of 173 sheep (*Ovis aries*), including both males and females aged 4 to 18 months, were screened for active and past CCHFV infections using RT-qPCR and enzyme-linked immunosorbent assay (ELISA). Of these, 104 CCHFV-negative sheep were randomly assigned to three experimental groups: an unvaccinated control group (*n* = 34), a group receiving the pDNA-HT vaccine (*n* = 35), and a group receiving the pDNA-CCHFV vaccine (*n* = 35).

Sheep were housed in open grazing systems under natural conditions, with supplemental feed provided as needed and ad libitum access to water. Immunizations were performed via intramuscular electroporation (IM-EP), with each animal receiving two injections of 1 mg plasmid DNA, 28 days apart. In this field study, all infections occurred through natural tick feeding, as sheep remained on extensive grazing land in an endemic area where exposure to *Hyalomma* and *Rhipicephalus* ticks is continuous throughout the year. No experimental challenge with CCHFV, WMV, or *R. conorii* was performed. All pathogen acquisitions reflected natural tick-borne transmission under typical husbandry conditions. Blood samples were collected at baseline, pre-boost, and post-vaccination time points, and sera were stored at −80 °C until further analysis. In addition to evaluating vaccine-induced immune responses, the study also assessed whether animals acquired WMV or *R. conorii* infections through natural field exposure, as described in detail in the pathogen-surveillance section below. Only sheep that tested negative for these pathogens at baseline were included. Unvaccinated control animals (*n* = 34) and animals vaccinated with pDNA-HT (*n* = 35) were monitored for new infections over the study period. Comparisons were made between these groups to determine if pDNA-HT reduced pathogen acquisition.

### Assessment of cross-protection against other tick-borne pathogens

Finally, we investigated whether the pDNA-HT vaccine could protect against other tick-borne pathogens (TBPs) unrelated to CCHFV, namely Wad Medani virus (WMV) and *Rickettsia conorii* (*R. conorii*). Both pathogens were selected because they are also prevalent in the same region in Senegal. In this field study, sheep were naturally exposed to circulating TBPs, and WMV and *R. conorii* were specifically chosen due to their known co-circulation in Northeastern Senegal and their transmission by tick species that frequently parasitize livestock in this area. Monitoring infection with these two pathogens, therefore, allowed us to assess whether the pDNA-HT vaccine could reduce the acquisition of additional TBPs under real-world natural exposure conditions.

Baseline blood samples were collected from all sheep to test for pre-existing IgG antibodies against WMV and *R. conorii* using ELISA. Animals seropositive prior to vaccination were excluded from further analyses to prevent confounding results. Subsequent blood samples were analyzed at 28-day intervals to track antibody responses post-vaccination.

### CCHF qRT-PCR

Viral RNA was extracted from sheep blood using the QIAamp Viral RNA Kit (Qiagen GmbH, Heiden, Germany). The extracted RNA was subjected to RT-qPCR assay, targeting the S-segment (N-gene) using the protocol described by Weidmann et al. ^36^. Briefly, the RT-qPCR reaction was performed using AgPath-ID One-step RT-PCR (Thermo Fisher Scientific), which includes both reverse transcription and PCR amplification in a single tube. The specific primers and probe used were:

- Forward primer (CCS FP): GGYACYAAGAAAATGAAGAAGG
- Probe (CCS P): CTGAGCACHCCAATGAARTGGGG
- Reverse primer (CCS RP): CRGGGAKTGTYCCRAAGCA

Thermal cycling consisted of reverse transcription at 50 °C for 10 min, initial denaturation at 95 °C for 10 min, followed by 45 cycles of 95 °C for 5 s and 60 °C for 15 s. For enhanced sensitivity, CCHFV detection was additionally performed using a touchdown profile, beginning with annealing temperatures decreasing from 70 °C to 64 °C in 2-degree steps (3 cycles each), followed by 33 cycles at 62 °C. Appropriate positive and negative controls were included in all runs to validate the result.

### ELISA

Mice and sheep serum samples were tested for IgG antibody by an enzyme-linked immunosorbent assay (ELISA) to assess the immune response. Plates were coated overnight with 50 ng of in-house–produced Bm86, CCHFV VLPs, CCHFV Gn protein (Cedarlane), CCHFV N protein (Abcam), or heat-inactivated antigen prepared from WMV-infected Vero E6 cell culture supernatant. WMV antigen was generated by infecting Vero E6 cells with the Wad Medani virus (strain ArD 398) and harvesting culture supernatants at the onset of cytopathic effect (CPE). Viral material was concentrated using PEG-8000 precipitation, resuspended in PBS, and heat-inactivated at 60 °C for 30 minutes. The antigen was then aliquoted and stored at −80 °C until use. For *R. conorii*, plates were coated with a commercial recombinant OmpA antigen (CUSABIO).

To differentiate antibody responses elicited by vaccination versus natural infection, IgG antibody against CCHFV N protein was used as a surrogate marker of natural infection, as the N protein was excluded from the vaccine formulation. Similarly, IgG responses against WMV and *R. conorii* were assessed using antigen-specific ELISA assays designed to detect infection-induced antibody responses.

Plates were washed with PBS containing 0.05% Tween-20 (PBS-T) and blocked with 5% non-fat milk prior to incubation with diluted animal sera. Of note, sheep serum samples were inactivated at 56 °C for an hour, diluted in blocking buffer, and tested at a 1:100 dilution. Following primary incubation, plates were washed and incubated with horseradish peroxidase (HRP)-conjugated secondary anti-mouse or anti-sheep IgG (Mandel Scientific, Guelph, Canada). After final washes, wells were incubated with 50 µl of 2,2’-azino-bis (3-ethylbenzothiazoline-6-sulfonic acid) (ABTS) substrate, and optical density (OD) values were read at 405 nm using a BioTek ELx808 microplate reader. Known positive and negative control sera were included in all assays to validate performance, and samples were tested in duplicate to ensure assay reliability.

### Statistical analysis

Statistical analysis was performed using GraphPad Software (version 9.4). Humoral responses were analyzed using one-way ANOVA followed by Dunnett’s test. Survival curves were compared using the log-rank (Mantel-Cox) test.

### Ethical statement

The sheep study was conducted in accordance with the Declaration of Helsinki and received approval from the National Ethical Committee for Health Research in Senegal (#00000806MSAS/DPRS/DR). The remaining animals have been integrated into the Pasteur Institute’s livestock herds at IPD, where they are maintained and may be utilized in other programs or to further the vaccine studies if needed.

Mouse experiments complied with the guidelines of the Canadian Council on Animal Care and were approved by the Animal Care Ethics Committee at Université Laval under research protocol number 2018042-1.

All experiments involving live vertebrate animals were conducted in accordance with relevant institutional and national guidelines and regulations. Animal husbandry conditions, inclusion criteria, randomization, anesthesia, euthanasia, and outcome assessment are described in the Methods section. Reporting of animal experiments was conducted in accordance with the ARRIVE Essential 10 guidelines.

## Data Availability

This study did not generate new sequencing, proteomics, structural, or genetic variation datasets requiring deposition in public repositories. All serological data supporting the findings of this study are available from the corresponding authors upon request.
